# NeoPeptide: an immunoinformatic database of T-cell-defined neoantigens

**DOI:** 10.1093/database/baz128

**Published:** 2019-12-09

**Authors:** Wei-Jun Zhou, Zhi Qu, Chao-Yang Song, Yang Sun, An-Li Lai, Ma-Yao Luo, Yu-Zhe Ying, Hu Meng, Zhao Liang, Yan-Jie He, Yu-Hua Li, Jian Liu

**Affiliations:** 1 Department of Hematology, Zhujiang Hospital, Southern Medical University, 253 Industrial Avenue, Haizhu, Guangzhou 510282, China; 2 School of Computer Science and Technology, Harbin Institute of Technology, 92 West Dazhi Street,Nan Gang District, Harbin 150001, China; 3 The Second Clinical Medical College (Zhujiang Hospital), Southern Medical University, 253 Industrial Avenue, Haizhu, Guangzhou 510282, China; 4 College of Computer Science, NanKai University, No.38 Tongyan Road, Jinnan District, Tianjin 300350, China

## Abstract

Therapeutic vaccines represent a promising immunotherapeutic modality against cancer. Discovery and validation of antigens is the key to develop effective anti-cancer vaccines. Neoantigens, arising from somatic mutations in individual cancers, are considered as ideal cancer vaccine targets because of their immunogenicity and lack of expression in normal tissues. However, only few databases support convenient access to these neoantigens for use in vaccines. To address this gap, we developed a web-accessible database, called NeoPeptide, which contains most of the important characteristics of neoantigens (such as mutation site, subunit sequence, major histocompatibility complex restriction) derived from published literature and other immunological resources. NeoPeptide also provides links to resources for further characterization of the novel features of these neoantigens. NeoPeptide will be regularly updated with newly identified and published neoantigens. Our work will help researchers in identifying neoantigens in different cancers and hasten the search for appropriate cancer vaccine candidates.

## Introduction

The ability of the immune system to recognize cancerous cells has opened the door for the development of cancer immunotherapy. Anti-cancer vaccines represent one of the most promising immunotherapeutic modalities against cancer. An ideal vaccine would harness the immune system for the selective destruction of cancer cells while having no adverse effect on normal tissues. Traditionally, anti-cancer vaccines have targeted tumor-associated antigens (TAAs), which are produced by genes overexpressed in cancer cells, such as Survivin (1), gp100 (2), WT1 (3), HER2/Neu (4) and MUC1 (5). These TAA-based vaccines have been shown to induce specific T responses *in vitro*; however, robust evidence of their long-term efficacy in cancer patients is yet to be obtained (6). A key limiting factor is that TAAs are also expressed in normal cells or germline tissues, although at low levels. This prevents an effective immune response due to central immune tolerance; contrarily, a strong immune response against these antigens may result in autoimmunity (7, 8).

**Figure 1:**
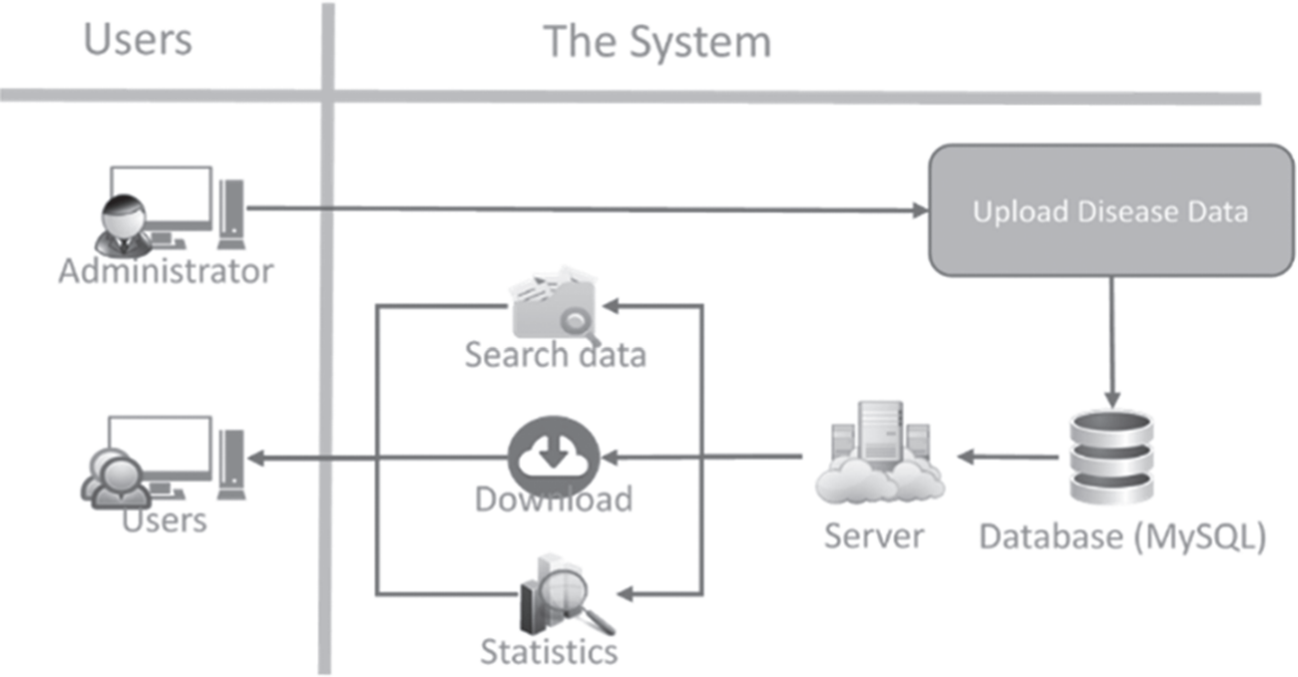
Structure of the neopeptide system.

Unlike TAAs, neoantigens, arising from somatic mutations, are expressed only on tumor cells (9). In this context, neoantigens may not be subject to immune tolerance and pose a lower risk of inducing autoimmunity; thus, these are considered as ideal cancer vaccine targets. However, translation of a somatic mutation into a neoantigen-based vaccine would entail at least four steps: (i) prediction of somatic nucleic acid exchange, (ii) identification of mutated proteins and the corresponding amino acid exchange, (iii) human leukocyte antigen (HLA) typing of patients and (iv) screening of the mutated peptide sequences with high affinity to HLA/MHC (major histocompatibility complex) molecules. The time required for these steps greatly reduces the work efficiency and limits the clinical utility (10–12). Therefore, there is a need to establish a database that catalogs the verified neoantigens and provides an effective search functionality to recognize neoepitopes as soon as a novel somatic mutation is defined (13–15). Previously developed databases have compiled a vast repertoire of traditional TAAs and largely ignored the importance of gathering epitopes derived from neoantigens [e.g. the AntiJen database (http://www.ddg-pharmfac.net/antijen/AntiJen/antijenhomepage.htm) (16)] or show the neoepitopes predicted from The Cancer Genome Atlas (TCGA) and the TCIA database (https://tcia.at/home) (17) [e.g. the TSNAdb database (http://biopharm.zju.edu.cn/tsnadb/) (18)] or are based on experimental data [e.g. the IEDB database (http://www.iedb.org/) (19)] or mostly focus on peptides identified previously [e.g. the CAPED database (https://caped.icp.ucl.ac.be/)]. Here, we introduce NeoPeptide, a web-based database, which provides access to search facility for neoantigens in the context of different cancers. NeoPeptide not only helps characterize the newly identified neoantigens (such as sequence, MHC restriction, adjuvant] but also provides additional information such as the corresponding nucleic acid and amino acid exchange in each neoepitope. In addition, NeoPeptide provides a way to further discover new characteristics of these neoepitopes.

## Materials and Methods

### Database implementation

The NeoPeptide database was built using tomcat 9.0.7 (web server) and MySQL (database server). All data were processed and organized into a MySQL database management system (version 5.7.20). The framework of the website was designed and implemented using the Java Spring MVC and the user interface was developed using html, css and jQuery libraries. The website has been tested using several web browsers, including Internet Explorer (e.g. version 11.648.17134.0), Google Chrome (e.g. version 73.0.3683.86) and Firefox (e.g. version 60.0.2).

**Figure 2:**
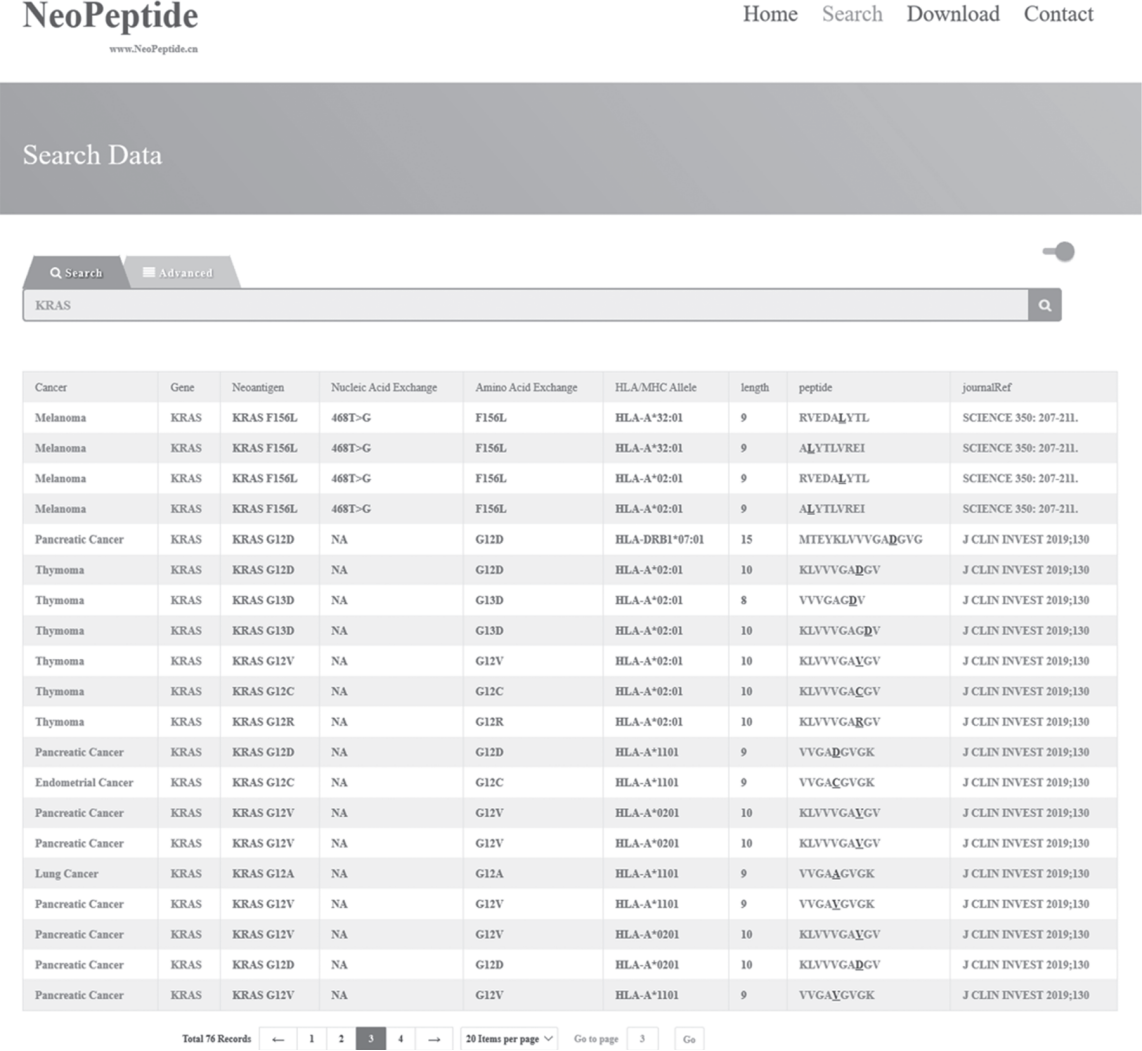
Search result example of ‘fuzzy search’. When the keyword ‘KRAS’ was searched, 76 entries were returned.

**Figure 3:**
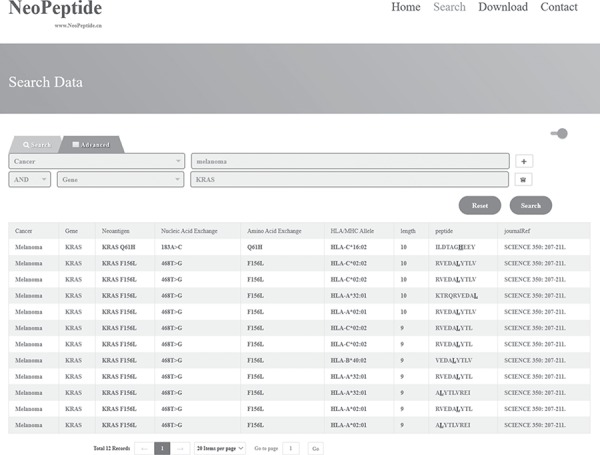
Search result example of ‘advanced search’. When the keywords ‘Melanoma’ and ‘KRAS’ were searched at the same time, 12 entries were returned.

### System architecture

A schematic illustration of the system architecture is shown in Figure 1. As this is a web-based application, users are not required to install complex software. In addition, a single browser is needed for searching and downloading the data.

### Search functionality

The search functionality has two modes. The first mode is the fuzzy search mode for general users, wherein users input a keyword to retrieve the related items. The web application sorts the retrieved items by priority, since the rules matching all fields may lead to a large amount of data. Therefore, we introduced the Levenshtein distance to help users quickly identify the data items of interest. The second mode is the advanced search mode wherein the advanced users can search for terms according to specific fields such as gene, neoantigen, amino acid exchange, HLA allele or peptide. Furthermore, there are three Boolean operators (AND, OR and NOT) to combine or exclude keywords for more focused results. The Boolean operator AND is the default mode; however, the users can alter the functionality using a pull-down menu.

### Download in NeoPeptide

The original data of NeoPeptide is freely available for download in the .dbf and *.xlsx formats. The .dbf file extension represents the dBase database file, and this format is supported by a number of database products. After downloading this file, users can import the data in their own database systems via the database import wizard function. For instance, users can use the database development tool Navicat (https://www.navicat.com/en/products/navicat-for-mysql), which incorporates the import wizard function for importing the .dbf file into a database system.

## Results

### Statistics of neoantigens in NeoPeptide

NeoPeptide is a comprehensive resource of somatic mutation-derived neoantigens. Neoantigens confirmed in cancer vaccines from clinical trials as well as *in vitro* experiments are recorded. Detailed information pertaining to the characteristics of the neoantigens is included in the database, such as HLA typing and corresponding nucleic acid and amino acid exchange.

NeoPeptide currently contains 181 137 epitopes derived from more than 36 000 neoantigens. These neoantigens have been defined in different cancers, including non-small cell lung cancer (e.g. *EGFR Q701L*), breast cancer (e.g. *TP53 S355F*), melanoma (e.g. *TMEM48 F169 L*), renal cell carcinoma (e.g. *ARMC8 T60I*), myeloproliferative neoplasms (e.g. *TET2 C784S*), gastric cancer (e.g. *BRCA2 S37 L*), uterine leiomyosarcoma (e.g. *SEC14L5 L289 V*), ovarian cancer (e.g. *ABCD2 E686Q*), pancreatic cancer (e.g. *MEN1 A68P*), colon cancer (e.g. *TP53 R175H*) and head and neck squamous cell carcinoma (e.g. *BRE S170 L*) and so on. In addition, NeoPeptide will be regularly updated to include newly identified and published neoantigens.

### Applications of NeoPeptide: a utility case

To facilitate database access, we developed a web interface that incorporates both simple and advanced search functionalities. For each entry, the main information includes ‘Cancer’ (the underlying disease from which the neoantigen was found), ‘Gene’ (the corresponding wild-type gene), ‘Neoantigen’, ‘Nucleic Acid Exchange’ (the nucleic acid somatic from the corresponding wild-type gene), ‘Amino Acid Exchange’ (the amino acid somatic from the corresponding wild-type gene), ‘HLA/MHC Allele’ (the HLA restriction of neoantigens used in the cancer vaccine), ‘Length’ (the length of neoantigen-derived epitopes used in the cancer vaccine), ‘Peptide’ (the sequence of neoantigen-derived epitopes) and ‘journalRef’ (publications that support the role of the neoantigen in cancer vaccine).

For demonstration purposes, the results of a fuzzy search ‘KRAS’ are shown in Figure 2. The database search retrieved 76 entries that have been experimentally identified or predicted as subunits in vaccines against melanoma, pancreatic cancer, thymoma or lung cancer. In the advanced search mode with restrictions of ‘melanoma’ and ‘KRAS’, 12 entries were retrieved (Figure 3). These results indicate that these 12 peptides may apply to neoantigen vaccine targeting the corresponding mutated KRAS and that this neoantigen vaccine may be effective in patients with melanoma. In addition, on clicking the entries in the ‘Cancer’ or ‘Gene’ column, the user will be directed to the NCBI’s search page (https://www.ncbi.nlm.nih.gov/search/) that contains detailed information about the corresponding cancer or the wild-type gene, respectively. Similarly, on clicking the entries in the ‘Peptide’ column, the user is directed to the IEDB webpage (http://tools.iedb.org/mhci/) for more details about the affinity to HLA or MHC molecules of these neoantigen-derived epitopes (Supplement information). Clicking the ‘journalRef’ link will navigate the user directly to the NCBI’s journal page.

## Discussions

Most cancers are characterized by a number of somatic mutations; however, not all of these mutations are suitable targets for cancer vaccine. According to the clinical trials of neoantigen vaccine, translation of somatic mutations into neoantigens for use in vaccines typically requires a period of 1–2 months. As most patients with advanced cancers survive for no more than 3 months, this time-consuming work represents a major barrier to the clinical use of neoantigen vaccines. Here, we developed a user-friendly neoantigen database, which may help researchers in quickly selecting the peptides for potential use in neoantigen vaccines. Moreover, NeoPeptide also provides useful links for further research on the functions of neoantigens. This database will be regularly updated with newly identified neoantigens in the context of a wider range of cancers. Further development of NeoPeptide will continue in order to offer a more comprehensive collection of experimentally supported data.

Many previous works mainly focus on the peptide information retrieval using the deterministic keyword inputs; this may affect the search capabilities and data FAIRness. Some databases (e.g. IEDB) raise concerns about ontology usage in recent years and make promising changes to reach the FAIR principles, which make it more interoperable with other projects. According to this, we will iteratively use ontology into the user interface, especially for disease term search and ‘HLA/MHC Allele’ search and reach more goals of FAIRness (19, 20).

## Supplementary Material

comments_-19_3_22_baz128

## Notes

**Database URL:** www.neopeptide.cn or https://github.com/lyotvincent/NeoPeptide
