# Supplementary material for: NeoPeptide: an immunoinformatic database of T-cell-defined neoantigens
Source: Database (Oxford). 2019 Dec 9;2019:baz128. doi: 10.1093/database/baz128 (PMC6901387; doi:10.1093/database/baz128)

**Supplement information**

NeoPeptide is linked to the active project IEDB. Since the IEDB web server does not support external link directly, unlike NCBI web server, users need to add the inputs manually. For convenience, we also develop a script based on the google chrome browser to help user add the inputs automatically. In particular, an extra script running on the user’s local browser needs to be installed. Firstly, a user opens the google chrome server and installs an extension named Tampermonkey at google web store (https://chrome.google.com/webstore/detail/tampermonkey/dhdgffkkebhmkfjojejmpbldmpobfkfo?utm_source=chrome-ntp-icon).


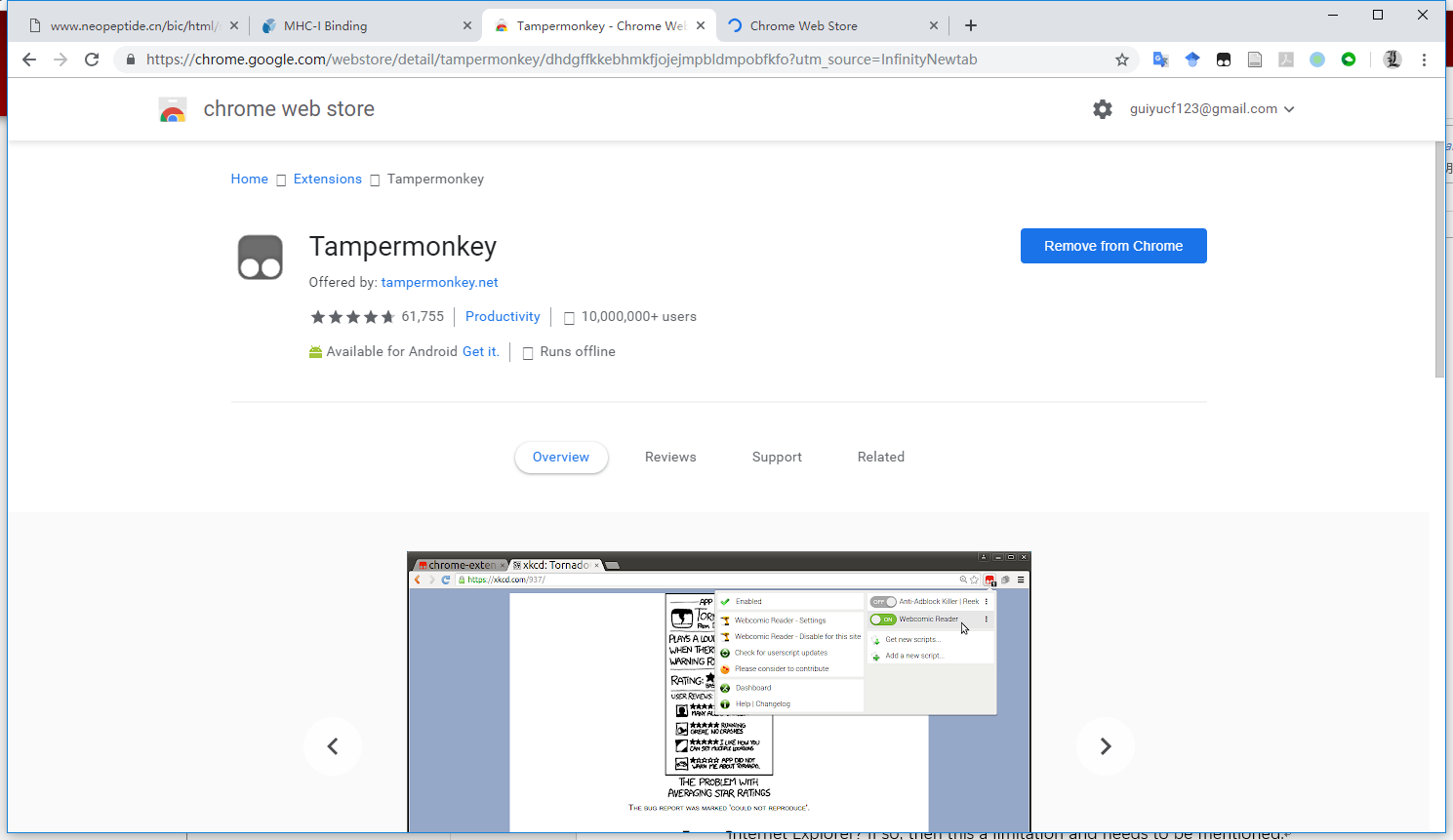


Secondly, a user uses chrome to open website <https://greasyfork.org/en/scripts/380715-iedb> for installing our script, and clicks "install this script".


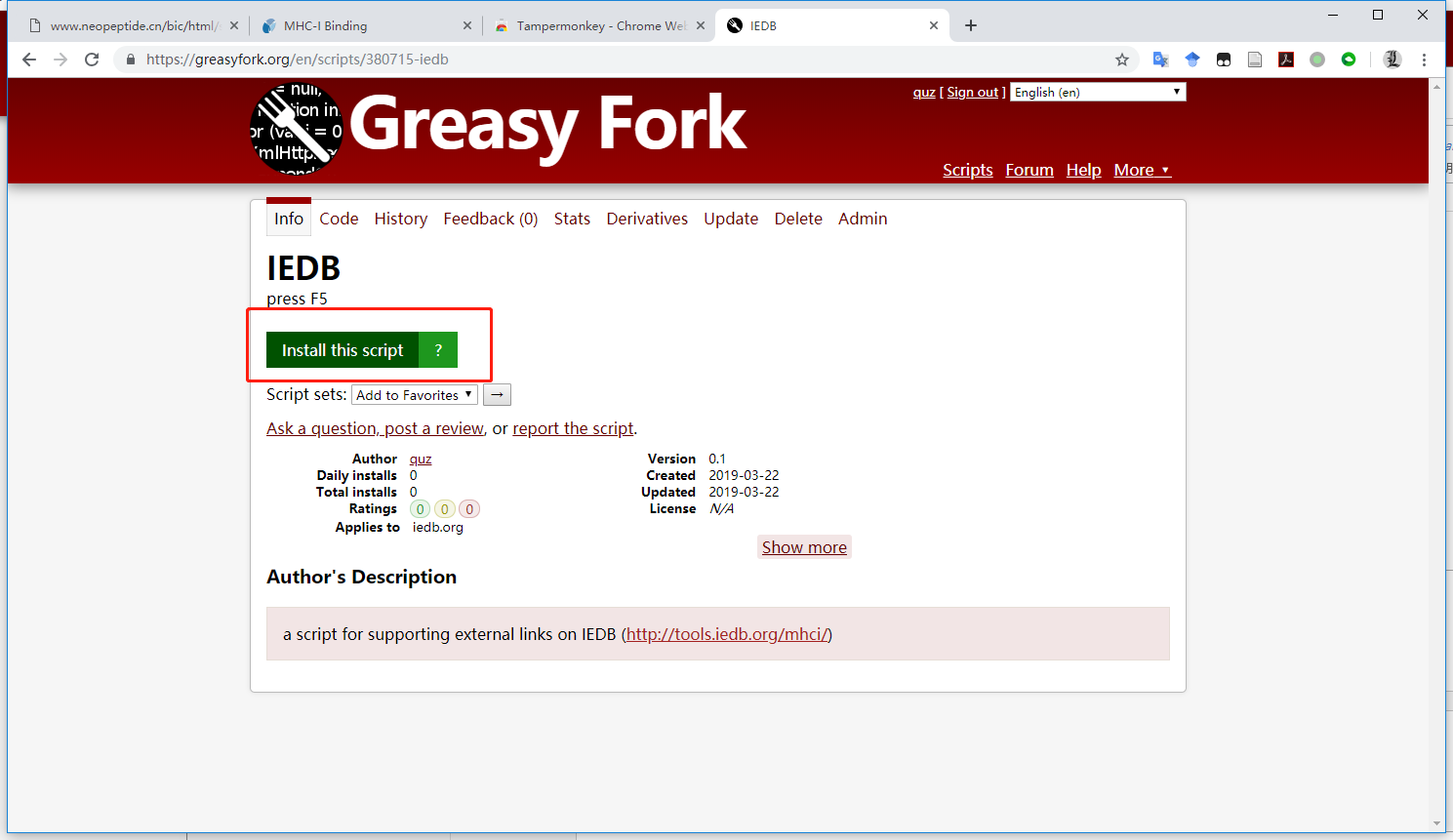


The new tab page will be shown in the following, and a user needs to click “install”.


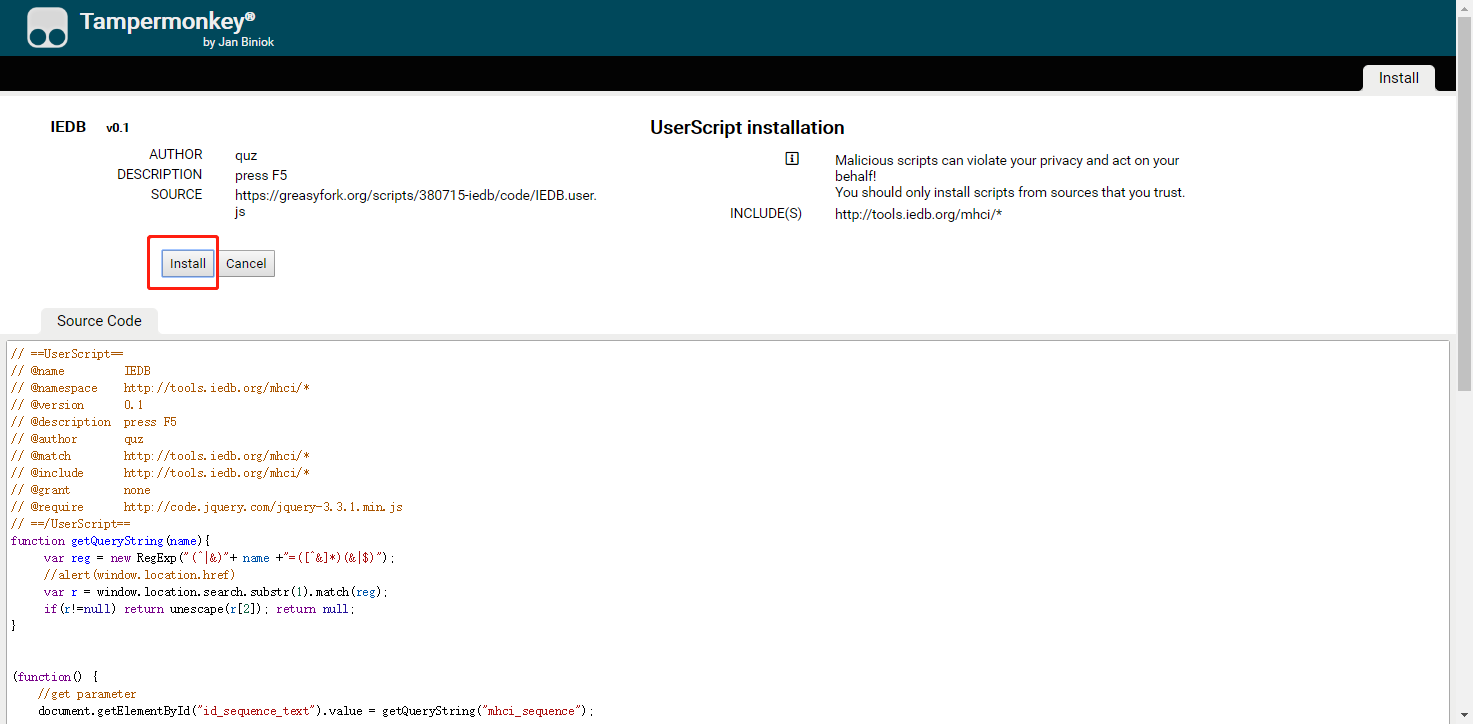


After the installation, user opens "Dashboard" of Tampermonkey, and clicks “IEDB”.


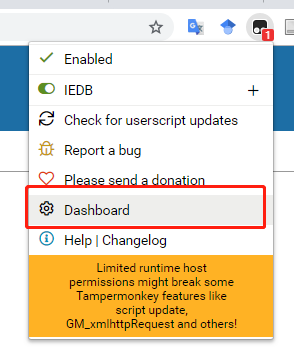


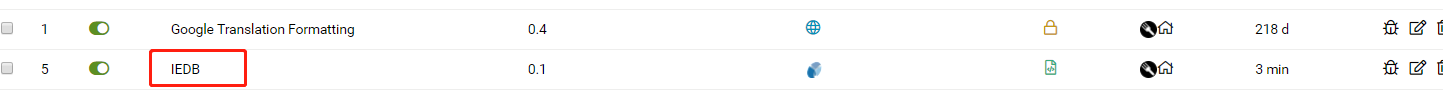


Next, choose "Settings" and select "document-body" at "Run at".


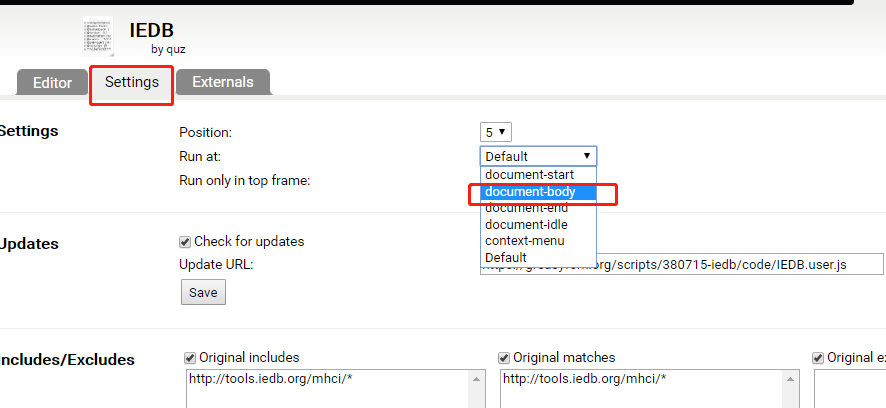


After finishing all the installation and the setting, user could add the inputs automatically and see the related results by clicking "submit" at IEDB.


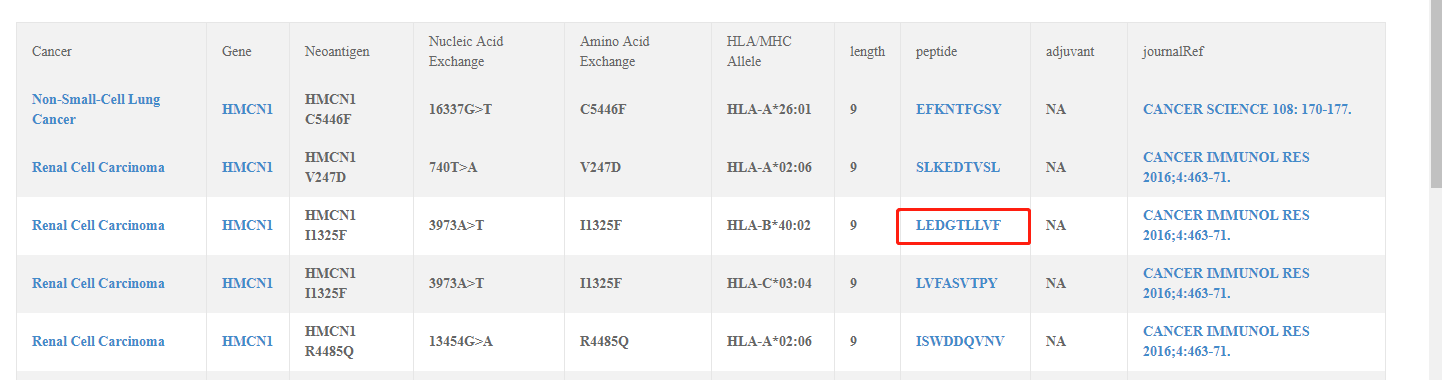


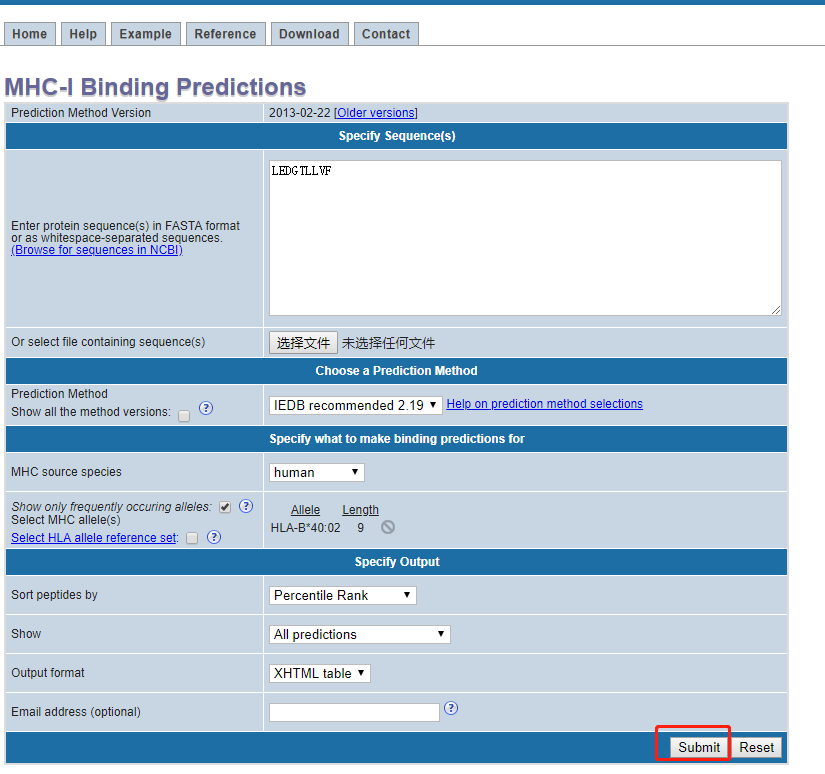

Supplement: comments_-19_3_22_baz128 [file comments_-19_3_22_baz128.doc]
